# Cu^0^-Functionalized, ZIF-8-Derived, Nitrogen-Doped Carbon Composites for Efficient Iodine Elimination in Solution

**DOI:** 10.3390/nano15020105

**Published:** 2025-01-12

**Authors:** Jiuyu Chen, Chensheng Gao, Jingwen Chen, Fei Liu, Zhiwen Liu

**Affiliations:** 1 School of Petroleum and Natural Gas Engineering, Changzhou University, Changzhou 213164, China; chenjy@cczu.edu.cn (J.C.); 19850300069@163.com (C.G.); 19103941135@163.com (Z.L.); 2 Key Laboratory for Protected Agricultural Engineering in the Middle and Lower Reaches of Yangtze River, Institute of Agricultural Facilities and Equipment, Jiangsu Academy of Agricultural Sciences, Ministry of Agriculture and Rural Affairs, Nanjing 210014, China; 3 State Key Laboratory of Lake Science and Environment, Nanjing Institute of Geography and Limnology, Chinese Academy of Sciences, Nanjing 210008, China

**Keywords:** ZIF-8, Cu^0^ nanoparticles, iodine, adsorption, DFT studies

## Abstract

The development of copper-based materials with a high efficiency and low cost is desirable for use in iodine (I_2_) remediation. Herein, Cu^0^-nanoparticles-functionalized, ZIF-8 (Zeolite Imidazole Framework-8)-derived, nitrogen-doped carbon composites (Cu@Zn-NC) were synthesized by ball milling and pyrolysis processes. The as-prepared composites were characterized using SEM, BET, XRD, XPS, and FT-IR analyses. The results showed that the morphology of ZIF-8 changed from a leaf-like structure into an irregular structure after the introduction of a copper salt and carbonization. The copper in the pyrolysis samples was mainly in the form of Cu^0^ particles. The presence of an appropriate amount of Cu^0^ particles could increase the specific surface area of Cu@Zn-NC. The subsequent batch adsorption results demonstrated that the as-fabricated composites showed high I_2_ adsorption amounts (1204.9 mg/g) and relatively fast dynamics in an iodine–cyclohexane solution when the Cu content was 30% and the pyrolysis temperature was 600 °C, outperforming the other Cu-based materials. The isothermal adsorption followed both Langmuir and Dubinin–Radushkevich isotherm models, while the kinetics of I_2_ adsorption followed a pseudo-second-order kinetic model. The activation energy (*E*_α_) of the adsorbent was determined to be 47.2 kJ/mol, according to the Arrhenius equation. According to the experimental and DFT analyses, I_2_-Zn interactions and I_2_-Cu^0^ chemisorption jointly promoted the elimination of iodine. In general, this study provided an operative adsorbent for the highly effective capture of iodine in solution, which might be worth applying on a large scale.

## 1. Introduction

Currently, diverse inorganic, organic, and metallic contaminants are inevitably generated in industrial processes [1,2,3]. The emission of pollutants could directly or indirectly enter water bodies, causing water pollution and creating a serious threat to the sustainable use of water resources while having a non-negligible impact on human health and ecosystems [2,4]. Therefore, water pollution has become an environmental problem that the world is facing at present. Iodine (I) is one of the pollutants that causes water pollution and is extremely undesirable due to its potential toxicity [1,5]. Its emission sources include iodine radioisotopes generated by nuclear fission in the process of nuclear energy utilization, wastes produced during water disinfection and purification, and wastes from scientific research institutions, among others [1,2]. Iodine mainly exists in the inorganic form (e.g., I_2_, I^−^, or IO_3_^−^) in wastewater [5]. The I_2_ species in the solution is easily assimilated by the human body via the food chain, which can have a negative impact on the normal metabolic processes [4,6,7]. Therefore, effective handling of iodine pollutants is essential to maintain the safety of ecological water environments and public health.

Surface adsorption is considered an economical and effective method for water pollutant treatment [8,9,10]. In this approach, various materials, such as carbon-based materials [11,12,13], covalent organic frameworks (COFs) [14,15], aerogels [16,17], and metal oxide-based materials [18,19,20], have been designed and applied to eliminate contaminants from aqueous solutions. Previous studies have suggested that adsorbents containing silver [21,22], copper [23,24], and bismuth [25,26] as the active adsorption components could enhance the interactions with iodine by providing valence electrons through π-complexation, thereby improving iodine adsorption efficiency [27]. In particular, silver-containing materials can outperform the other two metal-based composites in terms of equilibrium adsorption amount, stability, and adsorption kinetics for I_2_ capture [25,28]. Nevertheless, the toxicity and high cost of silver species restrict their large-scale application, rendering them impractical for utilization [29]. Cu-based materials have several advantages over silver-based materials, including a lower cost, less toxicity, and greater usability. Additionally, metallic copper or cuprous materials can react with I_2_ to form thermodynamically stable compounds (CuI), which are beneficial for the subsequent immobilization and stable storage of iodine-containing waste [8,30]. Therefore, more research has begun to explore strategies to improve the stability and iodine removal activity of Cu-based composites. To date, various Cu-based adsorbents, including Cu/MIL-101 [23], Cu-loaded zeolites [31], Cu^0^-SBA-15 [24], GA-Cu-ED [32], Cu^2+^-MOF-303 [30], and Cu/ZnO@C [8], have been synthesized and showed relatively high I_2_ adsorption capacities. For example, He et al. [24] prepared Cu^0^-SBA-15 with high thermal stability and applied it to eliminate I_2_. The batch adsorption test showed that the I_2_ adsorption capacity of Cu^0^-SBA-15 reached 842 mg/g. Miensah et al. [8] discovered that Cu/ZnO@C materials had an outstanding I_2_ capture capacity (1208 mg/g). However, they had a slow adsorption rate, and approximately 15 h was required to reach equilibrium adsorption. These results revealed that combining a high adsorption capacity with fast reaction kinetics is still a challenge for Cu-based adsorbents. Previous research has established that the uptake activity of a Cu-based adsorbents is connected to both the active sites and the support materials [29,33]. Therefore, further investigation into the influence of the substrate on the adsorption behavior is required in order to develop a strategy for designing Cu-based materials with a superior I_2_ removal ability.

Metal–organic frameworks (MOFs) are crystalline substances that are characterized by a topology resulting from the self-assembly of metal ions and organic ligands [30,34]. They exhibit benefits like elevated porosity, an extensive specific surface area, a low density, and diverse compositions. Based on these excellent properties, MOFs have found extensive applications, such as adsorption [19,23], catalysis [34], and storage applications [35]. Recent investigations have utilized MOF materials for iodine capture [10]. Although MOFs exhibit high iodine adsorption capacities, they are unstable under humid conditions and are prone to desorption after adsorption [36,37]. To address these aforementioned deficiencies, various carbon matrix-encapsulated active metal composites were prepared using MOFs as sacrificial templates [29,38]. The materials not only inherited the advantages of a large specific surface area and the porosity characteristic of MOFs, but this process also mitigated the drawback of inadequate stability in aqueous environments. Meanwhile, introducing active metals with a strong chemical affinity for iodine could also solve the issue of desorption after adsorption.

Inspired by this, herein, metallic-Cu-nanoparticles-decorated carbonaceous complexes (Cu@Zn-NC) were synthesized via pyrolysis of Cu-doped ZIF-8 precursors (Cu@ZIF-8). Thereafter, batch adsorption studies were conducted to assess the adsorption characteristics of the obtained composites based on initial I_2_ concentration, contact duration, and operational temperature. Simultaneously, the assessment of reusability was also conducted. The results of batch adsorption were integrated with density functional theory (DFT) theoretical calculations to systematically explore putative I_2_ removal mechanisms by the resultant composites.

## 2. Materials and Methods

### 2.1. Materials

Zinc nitrate hexahydrate (Zn(NO_3_)_2_·6H_2_O, Analytical Reagent, 99%), melamine (C_3_H_6_N_6_, Analytical Reagent, 99.5%), 2-Methylimidazole (2-MeIm, Analytical Reagent, 98%), cyclohexane (C_6_H_12_), absolute ethanol (C_2_H_6_O, Analytical Reagent, 99%), copper nitrate hydrate (Cu(NO_3_)_2_·3H_2_O, Analytical Reagent), and iodine (solid crystals, Analytical Reagent, 99.8%) were obtained from Shanghai Acmec Biochemical Technology Co., Ltd. (Shanghai, China) and Shanghai Aladdin Biochemical Technology Co., Ltd. (Shanghai, China), respectively. All aforementioned agents were of analytical purity and necessitated no additional purification.

### 2.2. Synthesis of ZIF-8 and Cu@ZIF-8

The synthesis process of ZIF-8 closely resembled that outlined in the literature [39]. In a typical synthesis, 100 mL of deionized water containing 3.3 g 2-MeIm and 1.2 g melamine was quickly mixed with another 100 mL of deionized water dissolved in 1.8 g Zn (NO_3_)_2_·6H_2_O. After stirring for 24 h at room temperature, the ZIF-8 precipitate was centrifuged, washed several times with ethanol, and dried in a vacuum at 60 °C overnight.

The Cu@ZIF-8 composite was typically fabricated using the following process: The prepared ZIF-8 powder (1.0 g) and different amounts of Cu (NO_3_)_2_·3H_2_O were ball-milled in a ball mill for 15 min at a speed of 300 r/min. Following this, the above samples were manually ground again for 15 min. The obtained product was labeled as xCu@ZIF-8, where x (x = 10%, 20% and 30%) was the mass ratio of Cu.

### 2.3. Synthesis of Cu@Zn-NC

**Figure 1:**
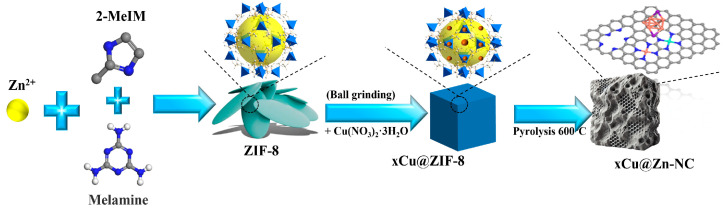
Schematic illustration of the synthesis procedure of xCu@Zn-NC samples.

The xCu@ZIF-8 precursor was subjected to pyrolysis in a tube furnace at 600 °C for 2 h in an argon environment, with a ramping rate of 5 °C/min. After the sample had naturally cooled to room temperature, it was collected. The obtained sample was designated as xCu@Zn-NC. For comparison, the Zn-NC product was also synthesized from the pyrolysis of ZIF-8 utilizing the same methodology. The synthesis protocol is illustrated in Figure 1.

### 2.4. Characterization

The morphology of the fabricated samples was characterized using scanning electron microscopy (SEM, JSM-IT500HR, JEOL Ltd., Akishima-shi, Japan). The Brunauer–Emmett–Teller (BET) surface area and pore size distributions were recorded using N_2_ adsorption–desorption isotherms (BSD-660, Beishide Instrument Technology (Beijing) Co., Ltd., Beijing, China). The phase and crystal structure were assessed with X-ray diffraction (XRD, Rigaku smartlab9, Rigaku Ltd., Akishima-shi, Japan). The composition and oxidation states of elements in the materials were examined via X-ray photoelectron spectroscopy (XPS, Thermo Kalpha, Thermo Scientific, Waltham, MA, USA). The surface functional group was measured using a Fourier transform infrared spectrometer (FT-IR, Nicolet IS50, Thermo Scientific, MA, USA).

### 2.5. Batch Adsorption Experiments

Batch adsorption tests, including isotherm adsorption, kinetics adsorption, thermodynamic adsorption, and reusability evaluation, were undertaken to assess the adsorption ability of the produced samples for I_2_. The detailed experimental procedure was as follows:

#### 2.5.1. Adsorption Isotherm Experiment

A total of 20 mg of the resultant composites was added to 20 mL of cyclohexane solution with I_2_ concentrations ranging from 300 to 2500 mg/L. The above turbid solution was stirred vigorously for 8 h. Then, 2 mL of supernatant was sampled and filtered with a 0.22 μm membrane filter. The I_2_ concentrations remaining in the diluted supernatant were detected using a UV–vis spectrophotometer at 523 nm.

#### 2.5.2. Adsorption Kinetic Experiment

The cyclohexane solution with an I_2_ concentration of 2500 mg/L was prepared, and then 200 mg of the resultant materials was added while stirring (stirring rate: 1200 rpm). After stirring for a certain period of time, 2 mL of the supernatant was sampled and filtered with the 0.22 μm membrane filter. The I_2_ concentrations remaining in the diluted supernatant were detected by UV–vis spectrophotometer at 523 nm.

#### 2.5.3. The Effect of the Solution Temperature

The cyclohexane solution with an I_2_ concentration of 2500 mg/L was prepared, and then 200 mg of a 30%Cu@Zn-NC sample was added into the above solution by stirring at 25, 35, 45, and 55 °C, respectively. After stirring for a certain period of time, 2 mL of the supernatant was sampled and filtered with the 0.22 μm membrane filter. The I_2_ concentrations remaining in the diluted supernatant were detected by UV–vis spectrophotometer at 523 nm.

#### 2.5.4. Regeneration and Reusability Evaluation

The adsorbents after saturated adsorption were collected and dried under vacuum at 65 °C for 12 h. Then, the post-adsorbed material was calcined at 600 °C for 2 h under argon atmosphere. Reusability tests were conducted through adsorption–regeneration steps.

### 2.6. Theoretical Simulation Calculation

A density functional theory (DFT) calculation was conducted to examine the interactions between the fabricated materials and I_2_ using the Materials Studio 2020 and Vienna First Principles Simulation Package (VASP 6.1.0). The Perdew–Burke–Ernzerhof (PBE) functional in the generalized gradient approximation (GGA) was used to describe the electron–ion interaction. The cutoff energy of the plane wave basis was set to 400 eV, and the gamma point was used to sample the first Brillouin zone. The accuracy of the electronic self-consistency was set to 10^−5^ eV between two electronic steps. The structure was completely relaxed by converging the residual forces between atoms below 0.001 eV/Å. In addition, a vacuum layer with a thickness greater than 15 Å was added to prevent interference during the calculation process. Finally, the Gaussian tailing method with σ = 0.1 eV was used to better assist the electronic convergence. The definition of the adsorption energy (*E*_ads_) was as follows:

(1) $$E_{ads}=E_{adsorbent+I_{2}}-(E_{adsorbent}+E_{I_{2}})$$

where *E*_adsorbent+I2_, *E*_adsorbent_, and *E*_I2_ represented the total energy of I_2_ adsorbed on the adsorbent surface, the unoccupied adsorbent surface, and I_2_ compounds, respectively.

## 3. Results and Discussion

### 3.1. Materials Characterization

**Figure 2:**
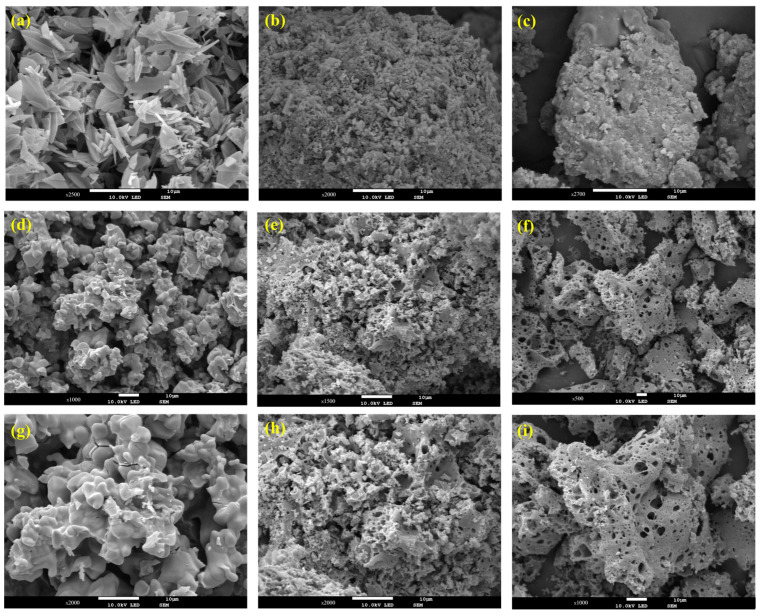
(**a**–**c**) SEM images of ZIF-8 (**a**), 20%Cu@ZIF-8 (**b**), and 30%Cu@ZIF-8 (**c**); (**d**–**i**) SEM images of Zn-NC (**d**,**g**), 20%Cu@Zn-NC (**e**,**h**), and 30%Cu@Zn-NC (**f**,**i**) in different magnifications.

The morphologies of the samples were characterized using SEM. As presented in Figure 2a, ZIF-8 particles exhibited a smooth surface and leaf-like morphology with length and thickness at the micrometer and nanometer levels, respectively. After copper introduction, the leaf-like morphology of the ZIF-8 sample disappeared and was replaced by an irregular morphology with a layer of flocculent structure covered on the surface (Figure 2b,c). Upon pyrolysis, the morphology and structure changed. As shown in Figure 2d,g, the leaf-like structure of ZIF-8 collapsed due to its inferior mechanical stability, resulting in a polyhedron with random distribution and irregular shape. This change indicated that the organic ligand of ZIF-8 might decompose during high-temperature calcination, resulting in the collapse of the crystal structure and the formation of a structure with more pores. These pore structures were conducive to increasing the specific surface area, potentially improving its performance in catalytic or adsorption processes [38,40]. In the case of xCu@Zn-NC (Figure 2e,f,h,i), a pronounced porous structure was observed, and the pore size increased with the increasing Cu content. The elevated content of Cu species (nitrate) might exacerbate the disintegration of ZIF-8 during pyrolysis, intensifying structural collapse and thus forming a large number of macroporous structures [29,38].

**Figure 3:**
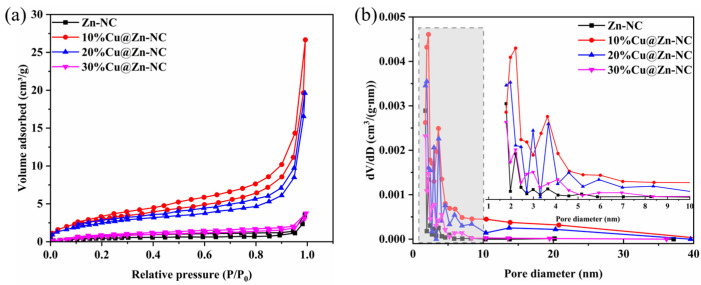
N_2_ adsorption–desorption isotherms (**a**) and pore size distributions (**b**) of xCu@Zn-NC samples. (Gray area: the zoom position in the diagram).

The specific surface area and pore structure of Zn-NC and xCu@Zn-NC samples were tested by the N_2_ adsorption–desorption analysis. Figure 3 demonstrated that all samples displayed type IV isotherms, signifying that the porous structures were primarily mesoporous [29,41]. This observation could be further corroborated by the BJH pore size distribution curves, which showed the pore sizes of Zn-NC, 10%Cu@Zn-NC, 20%Cu@Zn-NC, and 30%Cu@Zn-NC samples were centered at around 2.23, 2.11, 2.12, and 2.32 nm, respectively. The BET surface areas of the samples were determined using N_2_ adsorption–desorption isotherms, and Table S1 provides a summary of the findings. Pure Zn-NC, 10%Cu@Zn-NC, 20%Cu@Zn-NC, and 30%Cu@Zn-NC were found to have BET specific surface areas of 1.98, 11.50, 9.66, and 1.50 m^2^/g, respectively. Obviously, the presence of an adequate amount of Cu particles could increase the specific surface area of xCu@Zn-NC. Nevertheless, the specific surface area decreased with the Cu content increasing. This could be attributed to the formation of larger particles during the carbonization process, as evidenced by the SEM results (Figure 2f,i).

**Figure 4:**
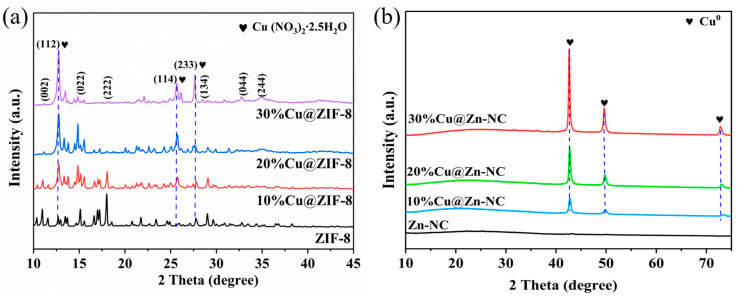
XRD patterns of xCu@ZIF-8 (**a**) and xCu@Zn-NC (**b**).

The phase composition and crystal structure of the composites were examined with XRD. The XRD spectra (Figure 4a) of the ZIF-8 sample showed sharp diffraction peaks at around 10.9°, 13.5°, 15.2°, 17.0°, 18.0°, 25.5°, 27.7°, 28.6°, 32.8°, and 34.9°, corresponding to (002), (112), (022), (013), (222), (114), (233), (134), (044), and (244) crystal planes, respectively. The relative intensities and prominent diffraction peak positions implied the formation of ZIF-8 crystals [42,43]. With the exception of the ZIF-8 crystals, several diffraction peaks ascribed to Cu(NO_3_)_2_·2.5H_2_O (JCPDS No. 75-1493) were also observed at around 13.5°, 25.5°, and 27.7° for xCu@ZIF-8 samples [44]. This could be the primary cause of variations in the diffraction intensity. After pyrolysis treatment, the characteristic diffraction peaks ascribed to ZIF-8 vanished, and a broadened derivative peak attributed to the (002) crystal plane of hexagonal graphitic carbon (JCPDS No. 89-8489) appeared at 2θ = 22.8° (Figure 4 and Figure S1), indicating that the organic matter in the precursor converted into the carbon matrix [3,45]. As for xCu@Zn-NC samples, three new sharp crystal diffraction peaks were detected at 2θ = 42.7°, 49.8°, and 72.8°, belonging to (111), (200), and (220) planes of Cu^0^ (JCPDS No. 04-0836), respectively. Additionally, as the percentage of Cu rosed from 10% to 30%, the intensity of the metallic Cu diffraction peaks increased.

**Figure 5:**
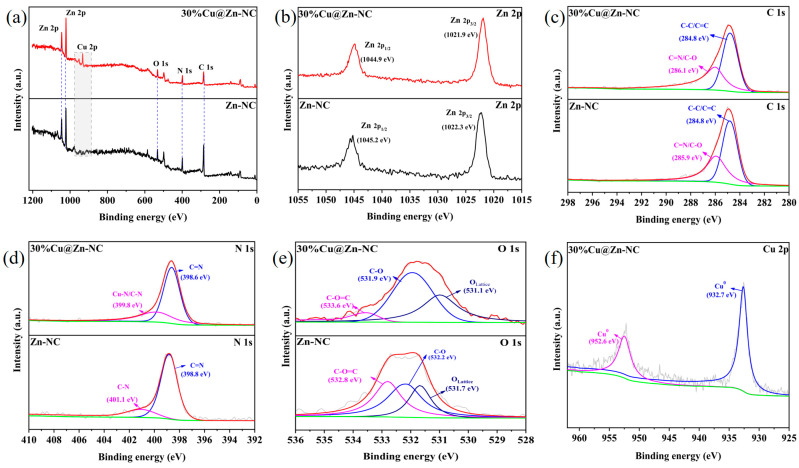
XPS patterns of Zn-NC and 30%Cu@Zn-NC. (**a**) Survey spectra; (**b**) Zn 2p spectra; (**c**) C 1s spectra; (**d**) N 1s spectra; (**e**) O 1s spectra; (**f**) Cu 2p spectra.

XPS research was performed to further examine the surface composition and oxidation state of elements in Zn-NC and 30%Cu@Zn-NC composites. Figure 5a and Table S2 confirmed the presence of Cu element in the 30%Cu@Zn-NC sample, while Zn, N, O, and C elements were distinctly observable in both samples. The Zn 2p XPS spectra (Figure 5b) for both samples had two peaks, which were attributed to Zn 2p_3/2_ and Zn 2p_1/2_, respectively. The Zn in Zn-NC and 30%Cu@Zn-NC was mostly present as Zn^2+^, as indicated by the binding energy difference of almost 23.0 eV between the two peaks [46]. Meanwhile, the analysis of the XRD pattern (Figure 4b) revealed the absence of diffraction peaks corresponding to Zn or ZnO, potentially indicating that the Zn species presented in an amorphous state in matrix [29]. The C 1s XPS spectra (Figure 5c) of the 30%Cu@Zn-NC sample could be deconvoluted into two peaks, representing the sp^2^-hybridized graphitic carbon C-C/C=C (284.8 eV) and C=N/C-O (286.1 eV) [3,47,48], respectively. Compared with 30%Cu@Zn-NC, the binding energy of C=N/C-O (285.9 eV) in Zn-NC had a slight shift (0.2 eV), which might be resulting from the incorporation of Cu. The N 1s XPS spectra (Figure 5d) could be deconvoluted into two peaks of C=N and C-N (C-N/Cu-N for the 30%Cu@Zn-NC sample), respectively. The presence of Cu-N peaks indicated that N could interact with atomically dispersed metal atoms to generate M-N_x_, while the shift of the binding energy of C-N/Cu-N peaks revealed N could influence the electronic characteristics of the carbon structure [3]. The O 1s XPS spectra (Figure 5e) was asymmetric and could be deconvoluted into three peaks, assigning to the C-O=C, C-O, and lattice oxygen, respectively. The Cu 2p XPS spectra (Figure 5f) in 30%Cu@Zn-NC exhibited two characteristic peaks of Cu 2p_3/2_ and Cu 2p_1/2_ at binding energies of 952.6 and 932.7 eV, respectively. No satellite peaks were found, suggesting that Cu in 30%Cu@Zn-NC existed as Cu^0^ or Cu^+^ [24,29,38]. Combining the XRD analysis, Cu in 30%Cu@ Zn-NC existed in the Cu^0^ form.

**Figure 6:**
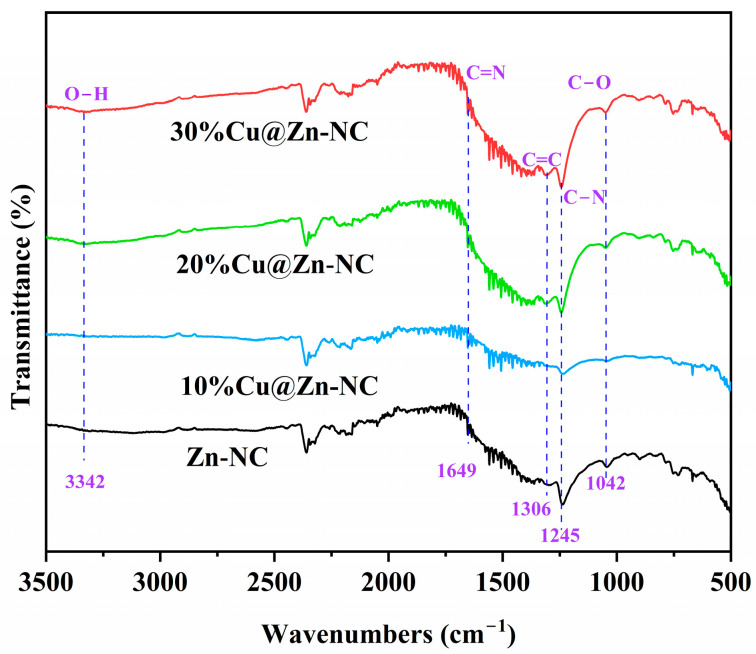
FT-IR spectra of xCu@Zn-NC samples.

FT-IR spectra was conducted to examine the functional groups in the xCu@Zn-NC samples (Figure 6). As depicted, the observed faint wideband at 3342 cm^−1^ corresponded to the stretching vibration of O-H, which resulted from the adsorption of ambient water molecules [38]. The bands around 1042, 1245 and 1306 cm^−1^ indexed to the stretching vibration of C-O, C-N and C=C bonds [49,50,51], respectively. The weak band at 1649 cm^−1^ was ascribed to the C=N stretching vibration [52]. These findings fundamentally corresponded with the results acquired from XPS analysis.

### 3.2. Batch Adsorption Performance

#### 3.2.1. Adsorption Isothermal Analysis

**Figure 7:**
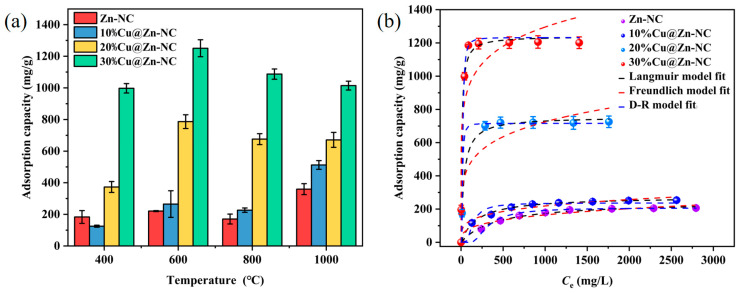
(**a**) The effect of Cu content and pyrolysis temperature on the I_2_ adsorption capacity; (**b**) adsorption isotherms of various samples for I_2_.

The influence of Cu proportion and pyrolysis temperature on the I_2_ adsorption performance of xCu@Zn-NC was investigated, and the result was shown in Figure 7a. As presented, the adsorption amount of the tested samples augmented with the increasing content of Cu at different pyrolysis temperatures. When the carbonization temperature increased from 400 to 1000 °C, the I_2_ adsorption performance of xCu@Zn-NC (x = 20% and 30%) initially increased and was followed by a subsequent decrease. When the Cu content was 30% and the pyrolysis temperature was 600 °C, the xCu@Zn-NC sample had the optimal adsorption capacity (1204.9 mg/g) for I_2_. Based on this, 600 °C was chosen as the pyrolysis temperature in subsequent research. Here, it should be noted that continuing to increase the Cu content in xCu@ZIF-8 would lead to a sharp decrease in the sample amount after carbonization. Therefore, considering the available sample amount and the adsorption performance, the Cu content ranged from 0 to 30%.

The equilibrium adsorption capacity of the as-made materials for I_2_ was studied by conducting adsorption isotherm experiments. As presented in Figure 7b, the equilibrium adsorption amount of all samples escalated significantly with the rise of the equilibrium concentration of I_2_. Furthermore, it could be further enhancement when Cu content increased. The saturated adsorption capacities of Zn-NC, 10%Cu@Zn-NC, 20%Cu@Zn-NC, and 30%Cu@Zn-NC for I_2_ were 206.4, 253.7, 725.6, and 1204.9 mg/g, respectively, much higher than that of the precursor (Figure S2). Obviously, the presence of Cu^0^ could significantly promote the adsorption performance of I_2_. This could be attributed to the addition of copper changed the surface charge of the adsorbent, as evidenced by the following charge density difference and Bader charge analysis.

The Langmuir, Freundlich, and Dubinin–Radushkevich (D-R) models were employed to analyze the isothermal uptake data of the synthesized adsorbent for I_2_. The Langmuir model assumed single-layer uniform adsorption, whereas the Freundlich model proposed multi-layer non-uniform distribution. The D-R model was suitable for determining whether the adsorption process was physical adsorption or chemical adsorption [53,54]. The nonlinear expressions of Langmuir (Equation (2)), Freundlich (Equation (3)), and D-R (Equation (4)) models could be described as follows:

(2) $$q_{e}=\frac{K_{L}\cdot q_{m}\cdot C_{e}}{1+K_{L}\cdot C_{e}}$$

(3) $$q_{e}=K_{F}\cdot C_{e}^{1/n}$$

(4) $$q_{e}=q_{m}\cdot \exp(-B\cdot (R\cdot T\cdot \ln(1+\frac{1}{C_{e}}{)}^{2})$$

where *q*_e_ and *q*_m_ (mg/g) represented the equilibrium adsorption capacity at equilibrium concentration *C*_e_ (mg/L) and theoretical maximum adsorption capacity, respectively. *K*_L_ denoted the Langmuir constant; *K*_F_ and n represented the Freundlich constant. *B* represented the constant associated with adsorption energy. *R* was the gas constant (8.314 J/mol·K), and *T* was the solution temperature (K). The fitting results were displayed in Figure 7b and Table S3. The correlation coefficients (*R*^2^) derived from both Langmuir model and D-R model exceeded those from the Freundlich model, indicating that the I_2_ capture by the adsorbent followed both Langmuir and D-R model. Furthermore, the *q*_m_ values (Table S3) of various samples determined by the Langmuir model and D-R model were mostly congruent with the isothermal adsorption data.

Beyond that, the average adsorption energy (*E*) of various samples was calculated by Equation (5):

(5) $$E=\frac{1}{\sqrt{2\cdot B}}$$

As shown in Table S4, the average adsorption energy of Zn-NC, 10%Cu@Zn-NC, 20%Cu@Zn-NC, and 30%Cu@Zn-NC was 2.6, 4.0, 11.8, and 14.7 kJ/mol, respectively. The value of *E* could be used to estimate the type of adsorption process. If the value of *E* was lower than 8 kJ/mol, the type of adsorption could be defined as physisorption, whereas for *E* value > 8 kJ/mol, the adsorption type was chemical adsorption [55]. Obviously, the I_2_ adsorption behavior by 20%Cu@Zn-NC and 30%Cu@Zn-NC was mainly governed by the chemical adsorption mechanism.

#### 3.2.2. Adsorption Kinetic Analysis

**Figure 8:**
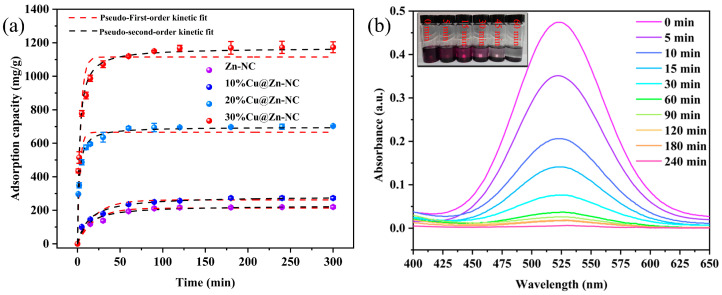
(**a**) Kinetic adsorption of I_2_ by xCu@Zn-NC samples, (**b**) UV–vis absorption spectra of I_2_ solution (900 mg/L) in different contact time.

The adsorption kinetic investigation was conducted to ascertain the adsorption rate and equilibrium time of the xCu@Zn-NC samples for I_2_. In Figure 8a, the adsorption amount of I_2_ by xCu@Zn-NC composites increased rapidly within the first 25 min. This might be characterized as an instantaneous adsorption stage or an external surface adsorption mechanism, attributable to the presence of adequate active adsorption sites on the xCu@Zn-NC surface [56,57]. However, as the contact time extended, the adsorption rate began to decrease because of the occupation of adsorption sites and the decrease of I_2_ concentration, making it difficult for the adsorbent to adsorb I_2_. Subsequently, a prolonged adsorption phase occurred, ultimately achieving equilibrium in roughly 60, 60, 30, and 30 min, with saturation uptake amounts of 219.5, 273.2, 703.1, and 1173.2 mg/g for Zn-NC, 10%Cu@Zn-NC, 20%Cu@Zn-NC, and 30%Cu@Zn-NC, respectively. To enhance the comprehension of the adsorption rate, the color change in the solution was documented at various time intervals. It could be clearly observed from Figure 8b that the solution color changed from dark purple to clear within 45 min. The UV–visible absorption spectra further validated that nearly all I_2_ in solution could be eliminated by the 30%Cu@Zn-NC composite. In pursuit of a more comprehensive investigation, the adsorption capability and adsorption kinetic of 30%Cu@Zn-NC toward I_2_ were evaluated in relation to other adsorbents documented in prior literature. The results (Table 1) showed that the uptake amount of 30%Cu@Zn-NC for I_2_ was significantly greater than or comparable, while it showed relatively fast adsorption kinetic.

To further reveal the rate-controlling step during the I_2_ removal process, the kinetic adsorption data were examined using the pseudo-first-order and pseudo-second-order kinetic models. The nonlinear expressions of the two models were as follows:

(6) $$q_{t}=q_{e}(1{-e}^{-k_{1}t})$$

(7) $$q_{t}=\frac{q_{e}^{2}k_{2}t}{1+q_{e}\cdot k_{2}\cdot t}$$

where *q*_t_ denoted the adsorption capacity at time *t* (min); *k*_1_ and *k*_2_ represented the adsorption rate constants for the pseudo-first-order and pseudo-second-order kinetic models, respectively. The fitting curves and the associated parameters were shown in Figure 8a and Table S5, respectively. According to the fitting curves and the *R*^2^ value, the adsorption kinetic data were more consistent with the pseudo-second-order kinetic model. Therefore, it could be inferred that the I_2_ adsorption behavior by xCu@Zn-NC was governed by the chemical adsorption mechanism, as evidenced by the following activation energy analysis.

#### 3.2.3. The Activation Energy Analysis

**Figure 9:**
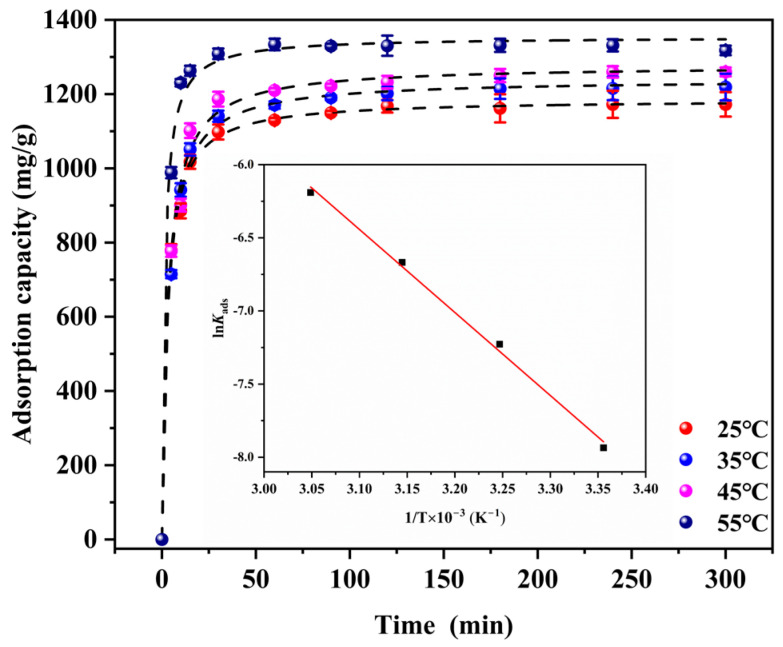
The effect of temperature on the I_2_ adsorption capacity of 30%Cu@Zn-NC sample. (Inset: Plot of ln *K_ads_* versus 1/*T*).

To reveal the impact of the solution temperature on the adsorption efficacy and the change in intrinsic energy during the adsorption process, the experiments on I_2_ adsorption by 30%Cu@Zn-NC were performed at temperatures of 25, 35, 45, and 55 °C, respectively. The findings (Figure 9) displayed that the equilibrium uptake capacity of 30%Cu@Zn-NC increased from 1172.3 to 1317.8 mg/g as the temperature elevated from 25 to 55 °C. Moreover, the capture rate was also greatly improved. When the solution temperature was 55 °C, the adsorption equilibrium was attained within 15 min. The adsorption thermodynamic data were analyzed using linearity expression of pseudo-second-order kinetic model, with findings presented in Table S6. According to the fitting results, the pseudo-second-order kinetic model more accurately represented the I_2_ removal behavior.

The activation energy was verified according to the Arrhenius (Equation (8)):

(8) ln⁡Kads=ln⁡A−EαR·T

where *E_α_* represented the activation energy (kJ/mol); *K_ads_* was the adsorption rate constant; *A* was the Arrhenius constant. From the pseudo-second-order kinetic studies (Table S6), *K*_s_ was the adsorption rate constant, i.e., *K_ads_*. According to previous research, the activation energy of physical adsorption was lower than 4.0 kJ/mol, while the activation energy of chemical adsorption was 8.5~83.5 kJ/mol [61]. In this work, *E_α_* was determined as 47.2 kJ/mol (Table S7), indicating that adsorption taken place through the chemical adsorption. Additionally, the positive value of *E*_α_ implied that increase in temperature favored the adsorption. Therefore, the endothermic characteristic of the adsorption process was indicated by the positive value.

#### 3.2.4. Regeneration and Reusability Performance

**Figure 10:**
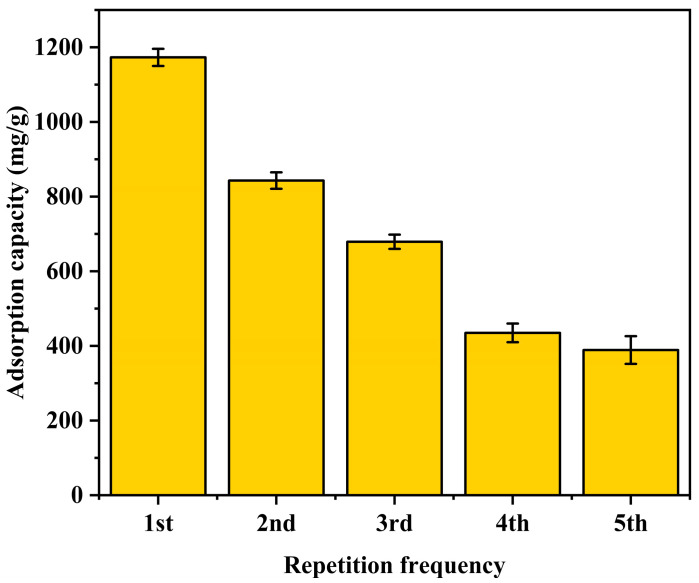
Reusability performance of 30%Cu@Zn-NC sample toward I_2_.

The regeneration and reusability performance of the 30%Cu@Zn-NC sample were investigated, with the findings illustrated in Figure 10. Following regeneration, the adsorption capability of the adsorbent diminished by approximately 30%, potentially attributable to the incomplete decomposition of CuI during the regeneration process. After five cycles, the adsorption amount measured 389.0 mg/g, representing merely 33% of the initial amount.

### 3.3. Adsorption Mechanism Analysis

**Figure 11:**
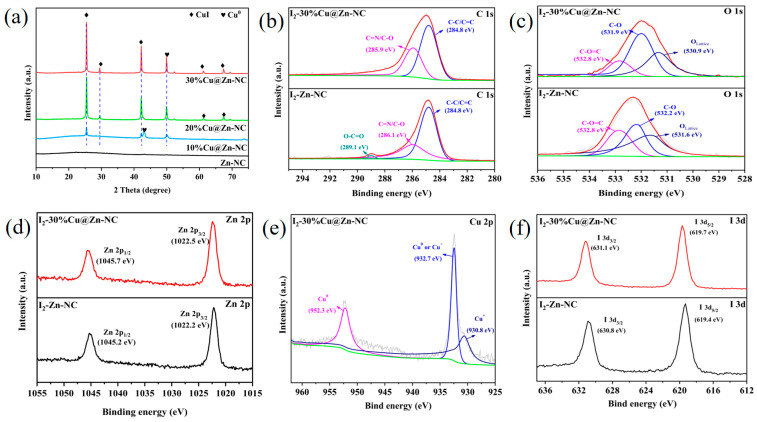
(**a**) XRD patterns of I_2_-NC and I_2_-x%Cu@Zn-NC. (**b**–**f**) XPS patterns of I_2_-Zn-NC and I_2_-30%Cu@Zn-NC. (**b**) C 1s spectra, (**c**) O 1s spectra, (**d**) Zn 2p spectra, (**e**) Cu 2p spectra, (**f**) I 3d spectra.

To achieve a comprehensive understanding of the I_2_ removal mechanism by the as-fabricated materials, XRD and XPS analyses were used to characterize the post-adsorbed composites (designated as I_2_-NC and I_2_-xCu@Zn-NC). Figure 11a demonstrated that the distinctive peaks corresponding to the CuI phase were detected for I_2_-xCu@Zn-NC at 2θ = 25.4°, 29.7, 42.2°, 61.3°, 67.6°, and 77.4° (JCPDS No. 06-0246), with the intensity of the diffraction peaks amplifying as the Cu proportion increased. Whereas the intensity of the Cu^0^ diffraction peak significantly decreased, indicating that Cu^0^ was crucial to the I_2_ elimination process. An XPS analysis was subsequently performed to ascertain the chemical structural information. The C 1s XPS spectra (Figure 11b) of I_2_-30%Cu@Zn-NC had two peaks, corresponding to the *sp*^2^ hybridized graphite carbon C-C/C=C (284.8 eV) and C=N/C-O (285.9 eV), respectively [29]. Compared with the pre-adsorption samples, the C=N/C-O (285.9 eV) peak showed a slight shift in energy levels (0.2 eV), which could be attributed to the interaction between C sites and I_2_ [11,62]. As for I_2_-Zn-NC sample, a new peak assigning to O-C=O appeared at binding energy of 289.1 eV. The O 1s XPS spectra (Figure 11c) of I_2_-30%Cu@Zn-NC revealed that the O 1s signal could be deconvoluted into three distinct peaks, representing C-O=C (532.8 eV), C-O (531.9 eV), and lattice oxygen (530.9 eV, Zn-O or/and Cu-O) [8,19]. The binding energy of the lattice oxygen peaks showed a shift after adsorption, which might be ascribed to the interaction between I_2_ and Zn or Cu sites, thus affecting the electron density, as illustrated in the following DFT analysis. The Zn 2p XPS spectra (Figure 11d) displayed two distinct peaks corresponding to Zn 2p_3/2_ and Zn 2p_1/2_, respectively. By comparing the characteristic peak binding energy of the samples before and after adsorption, it could be observed that the binding energy moved to a higher energy level post-adsorption. The reason might be that an inner sphere complex was formed between I and Zn sites (such as ZnI_2_) [38]. The Cu 2p XPS spectra (Figure 11e) of I_2_-30%Cu@Zn-NC displayed two characteristic peaks at binding energies of 952.3 and 932.7 eV, assigning to Cu 2p_3/2_ and Cu 2p_1/2_, respectively [29,38]. No satellite peaks were detected in the Cu 2p spectra, revealing the absence of Cu^2+^ species in I_2_-30%Cu@Zn-NC. And Cu was mainly found as Cu^0^ or/and Cu^+^ [8,24,63]. In conjunction with the XRD study (Figure 11a), it could be deduced that the Cu species in I_2_-30%Cu@Zn-NC existed as both Cu^0^ and Cu^+^ (CuI) states. According to the survey XPS scan (Figure S3a), two new distinct peaks appeared. The I 3d XPS spectra (Figure 11f) showed that the two peaks could be assigned to I 3d_3/2_ (631.1 eV) and I 3d_5/2_ (619.7 eV), respectively, confirming the adsorption of I_2_ on both samples [10,29].

**Figure 12:**
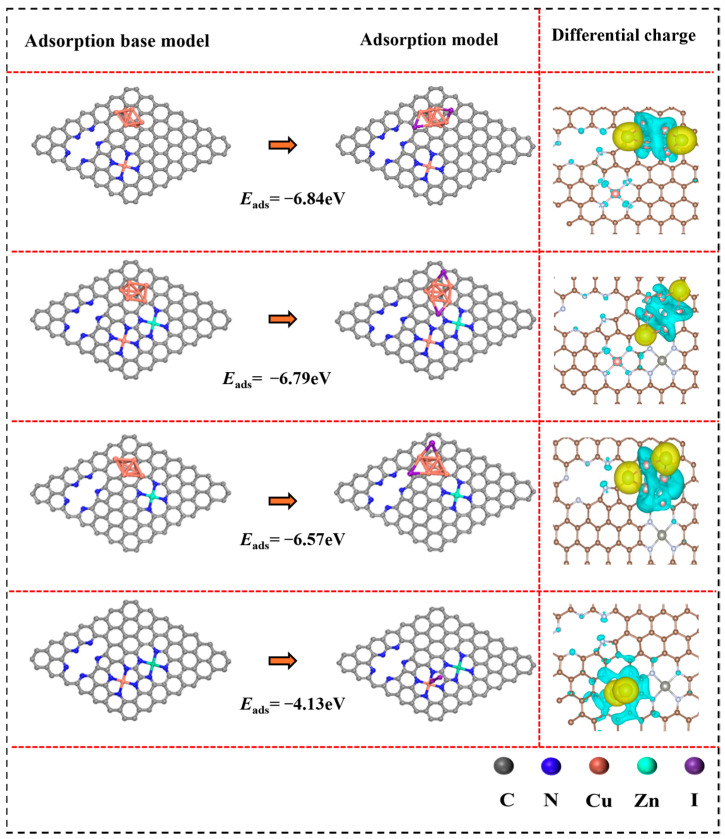
Configuration and charge density difference of I_2_ adsorption on different models of Cu@Zn-NC.

To better understand the interaction between the as-fabricated composites and I_2_, the adsorption energy and geometric states were investigated on several modeled structures by conducting DFT calculations. The optimal configurations of I_2_ molecules on various surfaces along with the accompanying adsorption energy were shown in Figure 12, Figures S4 and S5. As for the non-Cu-introduced configuration, the main adsorption sites included Zn, C, and N (Figures S4 and S5). Among them, N and Zn sites exhibited a greater affinity toward I_2_, and the adsorption energy of I_2_ on NC (C site), NC (N site), and Zn-NC (Zn site) was −1.45, −3.05, and −3.26 eV (Figure S4), respectively. Moreover, the molecular distance (Table S8) of the I-Zn bond was 2.673 Å, shorter than that of the I-C (3.912 Å) and I-N bond (2.914 Å). This demonstrated that Zn had a stronger affinity for I_2_ than C and N sites. Upon the incorporation of Cu (Figure 12), the optimized models were denoted as ZnNC (Cu)-I_2_, ZnCuNC (Cu)-I_2_, CuNC (Cu)-I_2_, and ZnCuNC-I_2_. The adsorption energy of the I_2_ on the Cu-containing configuration was approximately twice as high as that of the non-Cu introduced, indicating more potent chemical interactions occurred on the surface. The batch adsorption experiment would verify this conclusion. Especially when Cu was present in the carbon matrix as Cu clusters, the adsorption energy for I_2_ was higher than that of a single Cu site (ZnCuNC-I_2_). Meanwhile, by comparing the bond lengths of I-Cu in different models, the I-Cu bond length in ZnCuNC (Cu)-I_2_ was 2.5 Å (Table S8), shorter than in other modeled structures. From the aforementioned findings, we could infer that Cu clusters had a strong interaction with I_2_. The I-I bond could be cleaved at a specific density of the Cu sites, resulting in each iodine molecule being connected to two Cu sites. Chibani et al. and Chen et al. have obtained analogous results [10,40].

To intuitively demonstrate the charge density difference and electron transfer between the adsorbent and I_2_, the charge density difference and the Bader charge calculation analysis were conducted (Figure 12 and Table S9). The charge density difference and the Bader charge data showed that the iodine atoms in Cu incorporation models involved more charge transfer than the iodine atoms in non-Cu-introduced models or Cu existing in the form of Cu-N_x_. The aforementioned indicated that adsorption onto the Cu cluster arrangement included significant charge transfer, implying a strong affinity due to chemical interactions [64].

According to the above results, the elimination mechanism of xCu@Zn-NC toward I_2_ was mostly attributed to the robust chemical affinity of Cu^0^ to I_2_ (2Cu + I_2_ = 2CuI). In this process, the I-I bond could be cleaved, and each iodine atom could bond with Cu site and eventually form a stable CuI phase [6,16]. Additionally, Zn species might also act as the capture sites for I_2_ by forming an inner sphere complex (such as ZnI_2_) [38].

## 4. Conclusions

To summarize, this work presented the fabrication of xCu@Zn-NC composites by physical ball milling and pyrolysis process using inorganic Cu salts as raw sources and ZIF-8 as a precursor. The batch adsorption findings indicated that pyrolysis temperature, Cu ratio, and adsorption ambient temperature influenced the removal efficiency of the resulting materials. At a pyrolysis temperature of 600 °C and a Cu ratio of 30%, the sample exhibited a substantial I_2_ adsorption capacity of 1204.9 mg/g and an accelerated removal rate. Moreover, the adsorption amount and the equilibrium time could be further optimized when the I_2_-cyclohexane solution temperature was 55 °C. The exceptional I_2_ adsorption performance of the obtained materials was mainly ascribed to the strong chemical affinity of Cu^0^ nanoparticles for I_2_, as evidenced by analyzing the pre-/post-adsorbed materials, batch adsorption, and DFT calculation findings. The aforementioned results implied that the xCu@Zn-NC was an effective material for the removal of iodine from solutions. Prospectively, xCu@Zn-NC adsorbents were the potential candidate for efficient iodine removal form wastewater.

## Figures and Tables

**Table 1:** Comparison of I_2_ adsorption performance of 30%Cu@Zn-NC with other adsorbents reported in previous literature.

Adsorbent	Iodine State	*T* (°C)	Adsorption Capacity (mg/g)	Equilibration Time (h)	Reference
Cu/MIL-101	Gas/I_2_	75	432.0	24	[23]
Cu^0^-SBA-15	Cyclohexane/I_2_	RT	842.0	8	[24]
Cu/ZnO@C	Cyclohexane/I_2_	25	1280.1	15	[8]
Cu-MOF	Cyclohexane/I_2_	RT	971.0	10	[31]
Cu_2_O/Cu@C/SiO_2_	Gas/I_2_	150	820.0	2	[58]
XJU-1	Cyclohexane/I_2_	RT	368.0	48	[59]
ZIF-8@polyacrylonitrile	Cyclohexane/I_2_	RT	643.0	24	[60]
ZIF-8@wood	Gas/I_2_	75	1091.0	4.1	[40]
CZIF-1000	Cyclohexane/I_2_	RT	790.8	8	[10]
30%Cu@Zn-NC	Cyclohexane/I_2_	RT	1204.9	0.5	This work

## Data Availability

Data are contained within the article. The data presented in this study are available.

## Supplementary Materials

The following supporting information can be downloaded at https://www.mdpi.com/article/10.3390/nano15020105/s1: Figure S1: XRD patterns of Zn-NC and xCu@Zn-NC samples (10°–35°); Figure S2: The adsorption capacity of ZIF-8 and xCu@ZIF-8; Figure S3: XPS spectra of I_2_-Zn-NC and I_2_-30%Cu@Zn-NC, (a) survey spectra, (b) N 1s; Figure S4: Configuration and charge density difference of I_2_ adsorption on different models of Zn-NC; Figure S5: Sites distribution of various configuration, (a) NC-I_2_, (b) ZnNC-I_2_, (c) CuNC(Cu)-I_2_, (d) ZnCuNC(Cu)-I_2_, (e) ZnNC(Cu)-I_2_, (f) ZnCu-NC-I_2_; Table S1: BET surface aera, pore volume and average pore size of Zn-NC and xCu@Zn-NC samples; Table S2: Element content in 30%Cu@Zn-NC sample; Table S3: Fitting parameters of Langmuir, Freundlich and D-R isotherm models; Table S4: The average adsorption energy (*E*) of the adsorbent; Table S5: Kinetic parameters for the adsorption of I_2_ by xCu@Zn-NC nanocomposites; Table S6: Kinetic parameters for the adsorption of I_2_ by 30%Cu@Zn-NC nanocomposites in different solution temperature; Table S7: Fitting parameters of Arrhenius equation; Table S8: The bond length between I and the different adsorption sites; Table S9: Bader charge analysis.
