# Supplementary material for: Cu0-Functionalized, ZIF-8-Derived, Nitrogen-Doped Carbon Composites for Efficient Iodine Elimination in Solution
Source: Nanomaterials (Basel). 2025 Jan 12;15(2):105. doi: 10.3390/nano15020105 (PMC11767381; doi:10.3390/nano15020105)
Supplement: Supplementary file 1 [file nanomaterials-15-00105-s001.zip › nanomaterials-3370038-supplementary.pdf]

Supplementary materials

Cu^0-Functionalized, ZIF-8-Derived, Nitrogen-Doped Carbon Composites for Efficient Iodine Elimination in Solution

Jiuyu Chen [1], Chensheng Gao [1], Jingwen Chen [2,*], Fei Liu [3,*] and Zhiwen Liu [1]

[1] School of Petroleum and Natural Gas Engineering, Changzhou University, Changzhou 213164, China; chenjy@cczu.edu.cn (J.C.); 19850300069@163.com (C.G.); 19103941135@163.com (Z.L.)

[2] Key Laboratory for Protected Agricultural Engineering in the Middle and Lower Reaches of Yangtze River, Institute of Agricultural Facilities and Equipment, Jiangsu Academy of Agricultural Sciences, Ministry of Agriculture and Rural Affairs, Nanjing 210014, China

[3] State Key Laboratory of Lake Science and Environment, Nanjing Institute of Geography and Limnology, Chinese Academy of Sciences, Nanjing 210008, China

* Correspondence: chenjingwen@jaas.ac.cn (J.C.); feiliu@niglas.ac.cn (F.L.)

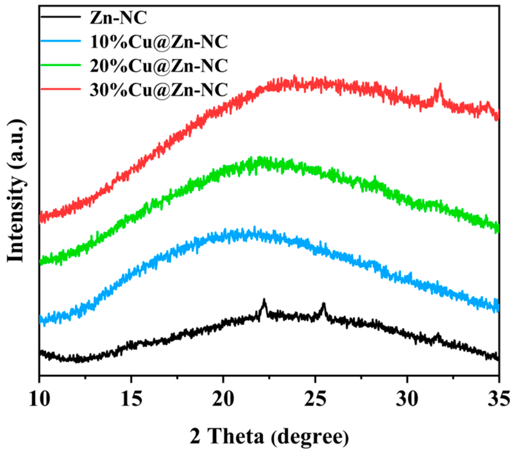

Figure S1. XRD patterns of Zn-NC and xCu@Zn-NC samples (10°-35°).

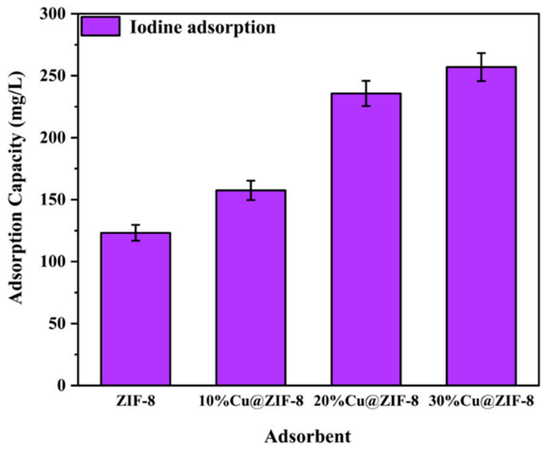

Figure S2. The adsorption capacity of ZIF-8 and xCu@ZIF-8.

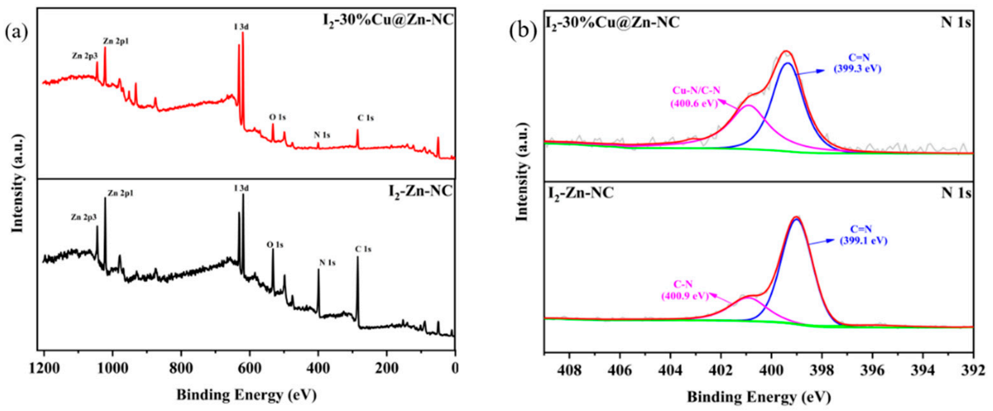

Figure S3. XPS spectra of I_2-Zn-NC and I_2-30%Cu@Zn-NC. (a) survey spectra, (b) N 1s.

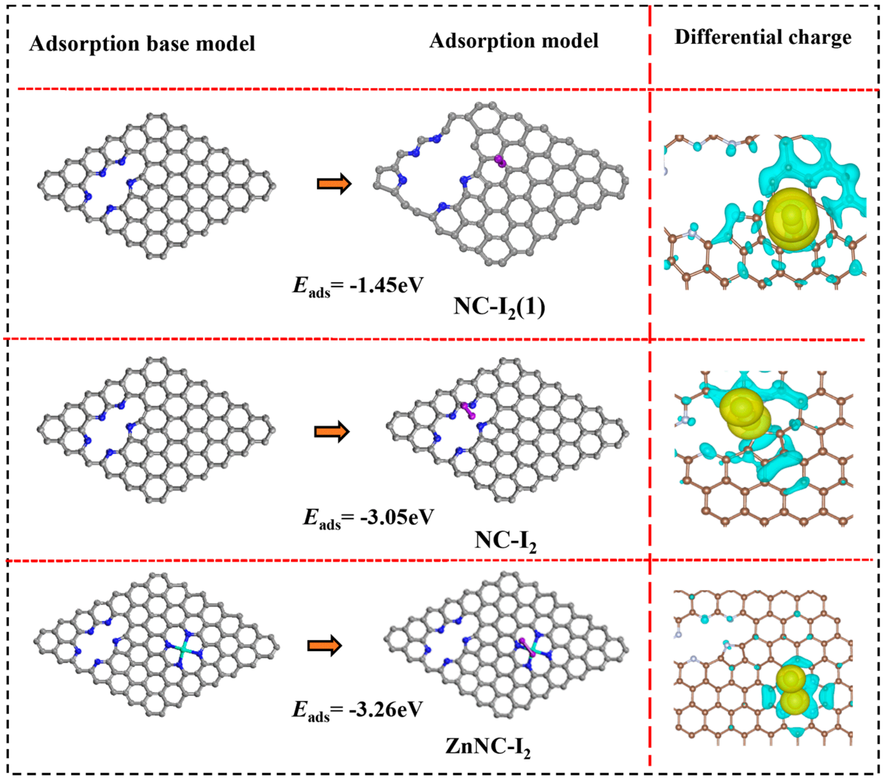

Figure S4. Configuration and charge density difference of I_2 adsorption on different models of Zn-NC.

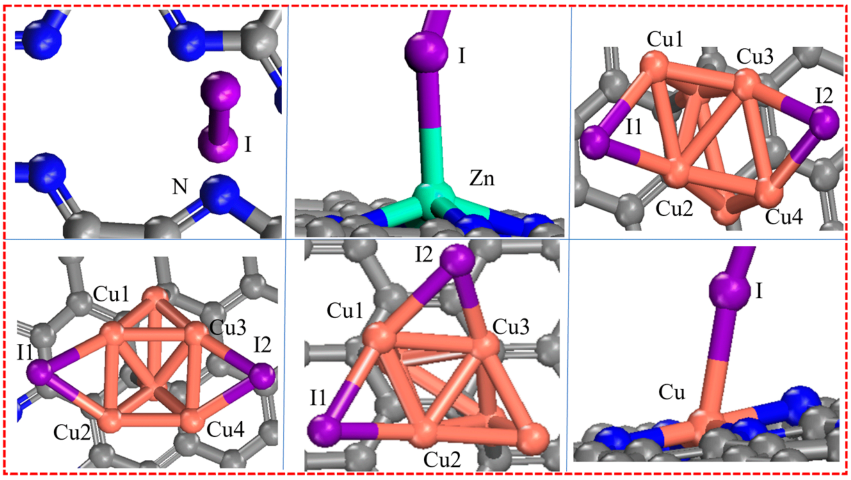

Figure S5. Sites distribution of various configuration. (**a**) NC-I_2, (**b**) ZnNC-I_2, (**c**) CuNC (Cu)-I_2, (**d**) ZnCuNC (Cu)-I_2, (**e**) ZnNC (Cu)-I_2, (**f**) ZnCu-NC-I_2.

Table S1. BET surface aera, pore volume and average pore size of Zn-NC and xCu@Zn-NC samples.

Adsorbent	BET surface aera (m^2/g)	Pore volume (cm^3/g)	Average pore size (nm)
Zn-NC	1.98	0.0051	6.98
10%Cu@Zn-NC	11.50	0.0393	9.56
20%Cu@Zn-NC	9.66	0.0286	10.10
30%Cu@Zn-NC	1.50	0.0057	5.23

Table S2. Element content in 30%Cu@Zn-NC sample.

Adsorbent	C	N	O	Zn	Cu
30%Cu@Zn-NC	50.65%	9.28%	30.65%	5.63%	3.79%
Zn-NC	66.20%	16.40%	9.40%	8.00%	—

Table S3. Fitting parameters of Langmuir, Freundlich and D-R isotherm models.

Adsorbent	Langmuir model			Freundlich model			D-R model		
	q_m (mg/g)	k_L (L/mg)	R^2	K_F	$1/n$	R^2	q_m (mg/g)	B	R^2
30%Cu@Zn-NC	1240.3	0.101	0.994	540.6	0.135	0.812	1232.3	0.0023	0.999
20%Cu@Zn-NC	755.3	0.029	0.996	194.8	0.191	0.891	716.1	0.0036	0.999
10%Cu@Zn-NC	275.1	0.005	0.995	42.8	0.236	0.909	237.1	0.0311	0.996
Zn-NC	245.9	0.002	0.991	21.1	0.298	0.947	207.5	0.0746	0.995

Table S4. The average adsorption energy (E) of the adsorbent.

Adsorbent	E (kJ/mol)
30%Cu@Zn-NC	14.7
20%Cu@Zn-NC	11.8
10%Cu@Zn-NC	4.0
Zn-NC	2.6

Table S5. Kinetic parameters for the adsorption of I_2 by xCu@Zn-NC nanocomposites.

Adsorbent	First-order kinetic equation			Second-order kinetic equation		
	q_e (mg/g)	k_1 (g/mg•min)	R^2	q_e (mg/g)	k_2 (g/mg•min)	R^2
30%Cu@Zn-NC	1114.8	0.269	0.943	1169.7	3.57×10^{-4}	0.991
20%Cu@Zn-NC	665.99	0.352	0.941	697.3	7.74×10^{-4}	0.989
10%Cu@Zn-NC	262.1	0.051	0.954	285.9	2.62×10^{-4}	0.986
Zn-NC	213.4	0.052	0.918	229.6	0.0514	0.957

Table S6. Kinetic parameters for the adsorption of I_2 by 30%Cu@Zn-NC nanocomposites in different solution temperature.

Temperature (°C)	Second-order kinetic equation		
	q_e (mg/g)	K_s (g/mg•min)	R^2
25	1185.9	3.58×10^{-4}	0.999
35	1240.3	3.61×10^{-4}	0.999
45	1277.7	3.83×10^{-4}	0.999
55	1354.3	2.05×10^{-3}	0.999

Table S7. Fitting parameters of Arrhenius equation.

Adsorbent	E_α (kJ/mol)	A	R^2
30%Cu@Zn-NC	47.2	68.87	0.991

Table S8. The bond length between I and the different adsorption sites.

Configuration		Molecular bond length (Å)
NC-I_2	I-N	2.914
	I-C	3.912
ZnNC-I_2	I-Zn	2.673
CuNC (Cu)-I_2	I1-Cu1	2.513
	I1-Cu2	2.573
	I2-Cu3	2.548
	I2-Cu4	2.508
ZnCuNC (Cu)-I_2	I1-Cu1	2.544
	I1-Cu2	2.523
	I2-Cu3	2.548
	I2-Cu4	2.530
ZnNC (Cu)-I_2	I1-Cu1	2.518
	I1-Cu2	2.506
	I2-Cu1	2.525
	I2-Cu3	2.512
ZnCu-NC-I_2	I-Cu	2.589

Table S9. Bader charge analysis.

Configuration	Electronic Transfer (eV)	
NC-I_2	+0.253	+0.233
ZnNC-I_2	+0.189	+0.207
CuNC (Cu)-I_2	+0.359	+0.358
ZnCuNC (Cu)-I_2	+0.3814	+0.383
ZnNC (Cu)-I_2	+0.242	+0.259
ZnCu-NC-I_2	+0.277	+0.289
